# A prospective observational study of drug therapy problems in medical ward of a referral hospital in northeast Ethiopia

**DOI:** 10.1186/s12913-018-3612-x

**Published:** 2018-10-22

**Authors:** Yaschilal Muche Belayneh, Gedefaye Amberbir, Asrat Agalu

**Affiliations:** Department of Pharmacy, College of medicine and Health Sciences, Wollo University, P.O. Box 1145, Dessie, Ethiopia

**Keywords:** Drug therapy problem, Dessie referral hospital, Hospitalized patients, Medical ward

## Abstract

**Background:**

Drug therapy problem is any undesirable event experienced by a patient during drug therapy that interferes with achieving the desired goals of therapy. Drug therapy problems are common causes of patient morbidity and mortality. There was no study that has been done on drug therapy problems in the study area, Dessie referral hospital, northeast Ethiopia.

**Method:**

A prospective observational study was conducted among hospitalized patients in the medical ward of Dessie referral hospital from March 01 to May 31, 2014. Ethical approval was obtained and informed consent was signed by each study participant before the commencement of the study. All patients admitted to the ward during the study period were included in the study. Data regarding each patient’s demographics, medical condition, drug therapy and patient compliance to the drug therapy were collected using pretested checklists, and drug therapy problems were determined based on the standard practice and textbooks. Descriptive statistical analysis was done using SPSS Version 20 Software.

**Result:**

A total of 147 patients were included, 75.51% of whom experienced at least one drug therapy problem. During the 3 month period a total of 159 drug therapy problems were identified of which needs additional drug therapy (35.85%) was the most common followed by unnecessary drug therapy (30.19%) and dosage too low (13.2%). Antibiotics, 75 (40.32%) was the most frequent drug class involved in drug therapy problems followed by cardiovascular drugs, 69 (37.1%) and nonsteroidal anti-inflammatory drugs, 9 (4.84%). Ceftriaxone (25.81%) was the most frequent specific drug prone to the drug therapy problems followed by spiranolactone (14.52%), enalapril (6.45%) and furosemide (6.45%).

**Conclusions:**

Three out of four patients experienced at least one drug therapy problem during their hospital stay in the medical ward, with the most commonly observed DTP being no drug therapy prescribed for a condition requiring drug treatment.

## Background

Drug therapy problem (DTP) is any undesirable event experienced by a patient related to drug therapy which interferes with achieving the desired goals of therapy. DTPs are usually classified as; needs additional drug therapy, unnecessary drug therapy, ineffective drug therapy, dosage too low, dosage too high, adverse drug reaction (ADR) and noncompliance. They are a major safety issue for hospitalized patients [1–3].

It is estimated that approximately 5 to 10% of all hospital admissions are drug related, and about 22% of patients are discharged with DTPs. As many as 28% of all emergency department visits are drug related [4, 5]. Common drug therapy problems resulting in emergency department visits are adverse drug reactions, noncompliance, and inappropriate prescribing [4]. Pharmaceutical drugs are associated with fatal adverse drug reactions in 3.1% of the total hospital admission and in 6.4% of those who die in hospital [6–8]. It has been estimated that 3 to 14% of total hospital admissions to medical wards are related to ADR which is one of the DTPs. ADR is one of the major challenges in the healthcare system due to increased patient morbidity, mortality, and healthcare costs [9–12].

According to the observational, longitudinal study at School Pharmacy of Newton Paiva University Center in Brazil, 91.7% of the patients experience at least one DTP during their hospital stay, 46.3% of patients experience more than three DTPs (ranging from 4 to 12 DRPs), 15.5% three DTPs and 11.3% two DTPs [13]. In Jimma University specialized hospital (JUSH) of southwest Ethiopia, unnecessary dug therapy was the most common drug therapy problem identified (24.2%) followed by needs additional drug therapy, 22.8%; noncompliance, 19.5% and dosage too low, 12.1% [14]. Generally DTPs are among the health care issues resulting in increased costs, morbidity and mortality when sever. To our knowledge, there was no previous study on drug therapy problems in hospitals of northeast Ethiopia particularly Dessie referral hospital. Thus, this study was aimed to determine the frequency of DTPs in the medical ward of Dessie referral hospital, northeast Ethiopia.

## Methods

### Study area and period

A prospective observational study was conducted from March 01, 2014 to May 31, 2014 in the medical ward of Dessie referral Hospital (DRH), located in Dessie town, northeast Ethiopia, 400 km from Addis Ababa. It is serving Dessie town and the surrounding population of about 7 million. This hospital is the only referral hospital in the northeastern part of Ethiopia. There were 165 health professionals working in the hospital. It has different Wards among which Medical ward is the one giving specialized medical services to the patients. There was no pharmaceutical care service in the hospital till the completion of this study.

### Study participants

A convenience sampling technique was employed to select patients for the study based on a study period. All patients admitted to the medical ward of the hospital during the study period, from March 01 to May 31, 2014, were included in the study. Critically ill patients requiring intensive care unit (ICU) admission and patients who were discharged before the collected data was crosschecked were excluded from the study.

### Data collection and identification of drug therapy problems

**Fig. 1:**
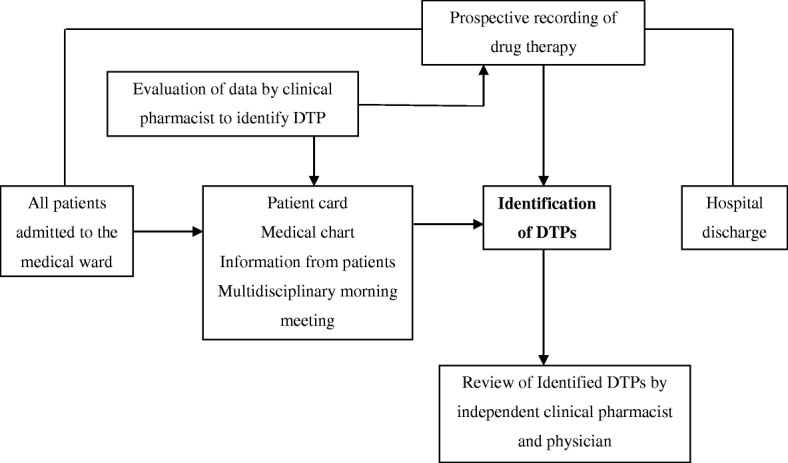
Flow chart for the prospective study of DTPs at the medical ward of DRH, March 01 to May 31, 2014

Data were collected by trained graduating class clinical pharmacy students using pre-tested data collection checklist. Data were collected from patient cards, medical charts, physicians’ ward rounds and the multidisciplinary morning meetings. The following data were recorded for each patient: age, gender, body weight, family and social histories, history of drug allergies, relevant medical and medication history, vital signs, drugs used at admission, drugs started during the hospital stay and at discharge, results of routine laboratory tests and the diagnosed diseases which are important for identification of drug therapy problems. The data collectors were involved in rounds of the medical ward to document each drug therapy, and each patient was assessed for compliance to the prescribed drug therapy using semi-structured interviews. Patient cards were reviewed, and patients were interviewed and followed starting from admission until discharge. Each documented drug therapy was evaluated for the presence of DTP everyday using standard textbooks and guidelines [15–20]. When a DTP was identified, the physicians were informed to resolve the problem for ethical reasons. The reliability and accuracy of each drug therapy problem was assessed by independent clinical pharmacist and physician. Unrealistic DTPs, which were not confirmed by both the pharmacist and physician, were excluded (Fig. 1).

The collected data were cleared and checked every day for completeness and consistency before processing. Data were entered and descriptive statistical analysis was done using SPSS Version 20 Software.

## Ethical consideration

Prior to the study, ethical approval was obtained from the ethical review committee of college of medicine and health sciences, Wollo University. The management of the Hospital was requested for cooperation with a formal letter from Wollo University. Each study participant signed a written consent to participate in the study. During and after the data collection process all patient-related data were kept confidential.

## Definition of terms

### Unnecessary drug therapy

A DTP that occurs when there is no valid medical indication for the drug at the time, or multiple drug products are used while only single-drug therapy is appropriate, or the condition is best treated with nondrug therapy, or the medical problem is caused by drug abuse, alcohol use, or smoking [1, 21].

### Needs additional drug therapy

A DTP that occurs when there is a medical condition needing new drug therapy, or preventive therapy is needed to reduce the risk of developing a new condition, or a medical condition requires combination therapy for better efficacy [1, 21].

### Ineffective drug therapy

The drug is not the most effective for the medical problem, or the drug product is not effective for the medical condition, or the condition is refractory to the drug product being used, or the dosage form is inappropriate [1, 21].

### Dosage too low

It is a DTP that occurs when the dose is too low to produce the desired outcome, or the dosage interval is too infrequent, or a drug interaction reduces the amount of active drug available, or the duration of therapy is too short [1, 21].

### Dosage too high

The dose is too high or the dosing frequency is too short or the duration of therapy is too long for the patient, or a drug interaction causes a toxic reaction to the drug product, or the dose was administered too rapidly [1, 21].

### Adverse drug reaction

The drug product causes an undesirable reaction that is not dose-related, or a safer drug is needed because of patient risk factors, or a drug interaction causes an undesirable reaction that is not dose-related, or the regimen was administered or changed too rapidly [1].

### Noncompliance

A DTP that occurs when the patient doesn’t understand the instructions, or the patient prefers not to take or forgets to take the medication, or the cost of drug product is not affordable for the patient, or the patient cannot swallow or self-administer the medication properly, or the drug product is not available for the patient [1, 21].

## Results

### Socio-demographic variables

One hundred forty seven (147) patients (age range, 18 to 74 years) were included in the 3 month study period; among which 87 (59.18%) were women. One hundred fourteen (114) of the patients (77.55%) were in the age range of < 50 years, 18 (12.24%) participants were in the age range of 50–64 and the rest 15 (10.2%) patients were in the age range of > 64 years. Of the total patients 90 (61.22%) were Muslims, 51 (34.69%) were orthodox Christians, 6 (4.08%) were protestant Christians. The majority of the analyzed patients were married, 99 (67.34%) (Table 1).

**Table 1:** Socio-demographic characteristics of the patients in the medical ward of DRH, March 01 to May 31, 2014 (*N* = 147)

Variable		N (%)
Sex	Female	87 (59.18)
	Male	60 (40.82)
Age	< 50	114 (77.55)
	50–64	18 (12.24)
	> 64	15 (10.2)
Education Level	Illiterate	69 (46.94)
	Primary school completed	24 (16.32)
	Secondary school completed	39 (26.53)
	Diploma	12 (8.16)
	Degree and above	3 (2.04)
Religion	Orthodox Christian	51 (34.69)
	Muslim	90 (61.22)
	Protestant	6 (4.08)
Marital status	Single	24 (16.32)
	Married	99 (67.34)
	Widow	3 (2.04)
	Divorced	21 (14.28)

### Drug therapy problems

All of the included patients had, at least, one disease with a diagnosis done by a physician. A total of 296 diseases were reported. Of the total patients 69 (46.94%) reported one disease, 44 (29.93%) reported 2 diseases, 15 (10.2%) reported 3 diseases, 7 (4.76%) reported 4 diseases, 7 (4.76%) reported 5 disease, 4 patients (2.72%) reported 6 disease and the remaining 1 patient (0.07%) reported 7 diseases. The most commonly reported diseases were cardiovascular disorders, 56 (18.92%) followed by GI disorders, 52 (17.57%); RTI, 48 (16.22%); CNS infections, 32 (10.81%) and hematologic disorders, 32 (10.81%) (Table 2).

**Table 2:** Frequency of diseases in the medical ward of DRH, March 01 to May 31, 2014

Diagnosed diseases	N (%)
Cardiovascular disorders	56 (18.92)
GI disorders	52 (17.57)
RTI	48 (16.22)
CNS infections	32 (10.81)
Hematologic disorders	32 (10.81)
Parasitic diseases	16 (5.41)
Endocrinologic disorders	16 (5.41)
Renal disorders	12 (4.05)
Opportunistic infections	8 (2.7)
RVI	8 (2.7)
Psychiatric disorders	8 (2.7)
Rheumatologic disorders	4 (1.35)
UTI	4 (1.35)
Total	296 (100)

Abbreviations: *GI* gastrointestinal, *RTI* respiratory tract infection, *CNS* central nervous system, *RVI* retroviral infection, *UTI* urinary tract infection

The average hospital stay of the patients in the medical ward of Dessie referral hospital was 6.2 days (range = 2 to 17 days). A total of 657 medications were prescribed for the studied patients during their hospital stay. The average utilization was 4.47 medications per patient (range = 2 to 9). The most commonly utilized medications were Antibiotics corresponding to 192 (29.22%) mentions (Table 3).

**Table 3:** Utilized medications in the medical ward of DRH, March 01 to May 31, 2014

Medications	N (%)
Antibiotics	192 (29.22)
Diuretics	132 (20.09)
Iron, Ca++, vitamins, and other supplements	60 (9.13)
NSAIDs	45 (6.85)
Antacids and antiulcer	45 (6.85)
Beta blockers	21 (3.2)
Antiretroviral drugs	18 (2.74)
Anti-malaria	15 (2.28)
Digoxin	15 (2.28)
Anticoagulants	12 (1.83)
ACEIs	12 (1.83)
Antifungal	9 (1.37)
Glucocorticoids	9 (1.37)
Insulin	6 (0.91)
calcium channel blockers	6 (0.91)
Propylthirouracil	6 (0.91)
Antiepileptic drugs	6 (0.91)
Antipsychotics	5 (0.76)
Direct vasodilators	5 (0.76)
Antihyperlipidemics	5 (0.76)
Oral hypoglycemic drugs	4 (0.61)
Antiarrhythmic drugs	4 (0.61)
Antiplatelates	4 (0.61)
Antihelmenthics	3 (0.46)
Others	18 (2.74)
Total	657 (100)

Abbreviations: *ACEI* angiotensin converting enzyme inhibitor, *NSAID* Nonsteroidal anti-inflammatory drug

Others: antiemetics, antidepressants, sedative-hypnotic drugs, antihistamines, acyclovir, Dextrometrophan, levodopa and carbidopa

A total of 159 DTPs were identified during the 3 month period involving 111 (75.51%) patients (69 females, 42 males) out of the 147 patients. An average of 1.08 drug therapy problems were recorded per patient, and an average of 0.48 DTPs were identified per medication order during the study period. The average incidence of DTPs was found to be 0.01/patient-day. Of the total patients, 69 (46.94%) had one DTP, 36 (24.49%) had two DTPs, 6 (4.08%) had 3 DTPs, and the remaining 36 (24.49%) had no identified DTPs.

**Fig. 2:**
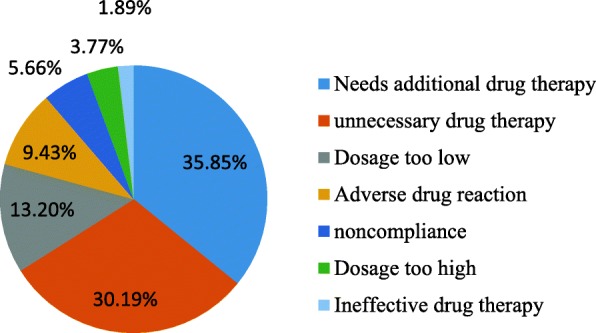
Identified drug therapy problems in the medical ward of DRH, March 01 to May 31, 2014

Of the 159 DTPs, needs additional drug therapy was the most common drug therapy problem identified accounting, 57 (35.85%). From the 57 DTPs classified as needs additional drug therapy, 33 (57.89%) were due to a medical condition indicated the need for initiation of drug therapy and 24 (42.11%) were due to the need for preventive drug therapy to prevent the development of a new medical condition. The second most common DTP was unnecessary drug therapy, 48 (30.19%). Of the 48 DTPs classified as unnecessary, 39 (81.25%) were due to the absence of valid medical indication at that time and 9 (18.75%) were due to duplication of therapy. The third most frequently identified drug therapy problem was dosage too low, 21 (13.2%). Other DTPs identified were ADR, 15 (9.43%); non compliance, 9 (5.66%) including the patient had no access to the medication 3 (33.33%) and the patient preferred not to take the medications 6 (66.67%); dosage too high, 6 (3.77%) including a drug-drug interaction resulted in a toxic reaction to the drug, 3 (50%), the duration of drug therapy was too long 3 (50%); ineffective drug therapy 3 (1.89%) (Fig. 2).

Analysis of drug classes involved in the drug therapy problems showed that antibiotics, 75 (40.32%), were the most frequent classes of drugs involved in the DTPs; followed by cardiovascular drugs, 69 (37.1%) involving diuretics, 40 (21.51%), ACEIs, 15 (8.06%), beta blockers, 12 (6.45%) and digoxin, 3 (1.61%) (Table 4).

**Table 4:** Classes of drugs involved in the identified DTPs in the medical ward of DRH, March 01 to May 31, 2014

Class of drugs	N (%)
Antibiotics	75 (40.32)
Diuretics	40 (21.51)
ACEIs	15 (8.06)
Beta blockers	12 (6.45)
NSAIDs	9 (4.84)
Iron, Ca++, vitamins and other supplements	9 (4.84)
Antihyperlipidemics	7 (3.76)
Dextromethrophan	6 (3.23)
Antiretroviral drugs	4 (2.15)
Antacid and antiulcer	4 (2.15)
Digoxin	3 (1.61)
Dextrometrophan	2 (1.06)
Total	186 (100)

Abbreviations: *ACEI* angiotensin converting enzyme inhibitor, *NSAID* Nonsteroidal anti-inflammatory drug

A total of 186 specific medications were involved in the identified drug therapy problems of all types. Ceftriaxone (25.81%) was the most frequent drug prone to the DTPs followed by spiranolactone (14.52%), enalapril (6.45%) and furosemide (6.45%) (Table 5).

**Table 5:** Specific drugs involved in the drug therapy problems in the medical ward of DRH, March 01 to May 31, 2014

Specific drug	N (%)
Ceftriaxone	48 (25.81)
Spiranolactone	27 (14.52)
Enalapril	12 (6.45)
Furosemide	12 (6.45)
Atenolol	6 (3.22)
Clarithromycin	6 (3.22)
Dexamethasone	6 (3.22)
Iron supplement	6(3.22)
Metronidazole	6 (3.22)
Propranolol	6 (3.22)
Others	51 (27.42)
Total	186 (100)

Others: Antibiotics, diuretics, ACEIs, BBs, NSAIDs, Antihyperlipidemics, Digoxin, Antiretroviral drugs, Ca++, vitamins and other supplements

The most common specific drugs involved in each particular DTP include spiranolactone (19.05%) as needed additional drug therapy, ceftriaxone (61.11%) as unnecessary drug therapy, and spiranolactone (28.57%) and ceftriaxone (28.57%) as dosage too low.

Most of the DTPs (41.5%) were occurred during the treatment of cardiovascular disorders followed by respiratory tract infection, 33 (20.75%); GI disorders, 27 (16.98%); CNS infection, 15 (9.43%); endocrinology disorders, 6 (3.77%); hematologic disorders, 6 (3.77%); RVI, 3 (1.89%) and OI, 3 (1.89%). Some of the identified drug therapy problems are mentioned below as example  (Table 6).

**Table 6:** Examples of drug therapy problems in the medical ward of DRH, March 01 to May 31, 2014

Type of DTP	Examples
Needs additional drug therapy	ACEI was not prescribed for AHA stage C, CHF with no contraindication to the medication. Propranolol or nadolol was not prescribed for the patient with chronic liver disease and portal hypertension to prevent variceal bleeding.
Unnecessary drug therapy	Dextrometrophan was prescribed for community acquired pneumonia. Ceftriaxone was prescribed with fluconazole to treat cryptococcal meningitis.
Dosage too low	Spiranolactone 50 mg PO once daily was prescribed to treat ascites instead of 100-400 mg per day. Ceftriaxone 1 g IV BID was prescribed to treat meningitis instead of 2 g BID.
ADR	Hypotension was developed due to furosemide 20 mg IV BID and spiranolactone 25 mg PO once daily.
Noncompliance	The patient preferred not to take enalapril which was prescribed to treat CHF.
Dosage too high	Digoxin 0.25 mg PO once daily was prescribed with Clarithromycin PO BID and then the patient developed cardiac arrhythmia.
Ineffective drug therapy	Atenolol was prescribed to treat AHA stage C, CHF.

Abbreviations: *PO* orally, *IV* intravenous, *BID* twice a day, *TID* three times a day, *AHA* American heart association, *CHF* chronic heart failure

## Discussion

This study has shown that 75.51% of the studied patients had DTPs. This is comparable to the result of the study in Norway which reported 81% of the hospitalized patients had DTPs [22]. In this study an average of 1.08 DTPs were recorded per patient. This is also comparable to the result of the study at the medical wards of Grenoble University Hospital in which an average of 1.71 DRPs were identified per patient [23]. But according to a retrospective study in Australia, 4.6 drug therapy problems were identified per patient [24]. This is high as compared to the results of our study and the reason may be due to lower number of diagnosed diseases per patient during the study period which can possibly lead to decreased occurrence of DTPs in the medical ward of DRH.

Of the 159 DTPs identified in this study, needs additional drug therapy (35.85%) was the most common drug therapy problem; followed by unnecessary drug therapy, 30.19%; and dosage too low, 13.2%. This is comparable to the result of a prospective observational study which was conducted in JUSH in which unnecessary dug therapy was the most common drug therapy problem identified (24.2%) followed by needs additional drug therapy, 22.8%; noncompliance, 19.5% and dosage too low, 12.1% [14]. Noncompliance accounts for 5.66% of the total identified DTPs in our study which was lower compared to the result of the study in the medical ward of JUSH (19.55%) [14]. This difference might be due to the lower average utilization of medication per patient in the medical ward of DRH which can possibly reduce the occurrence of patient noncompliance.

Antibiotics were the most frequent classes of drugs involved in DTPs, 40.32%; followed by cardiovascular drugs, 37.1%; NSAIDs, 4.84%; iron, Ca++, vitamins and other supplements, 4.84%. This result is similar to the study in the medical wards of Grenoble University Hospital in which cardiovascular drugs were the most frequently implicated (22.2%), followed by antibiotics/anti-infective (13.3%) and analgesics/anti-inflammatory drugs (11.3%) [23].

According to this study ceftriaxone (25.81%) was the most frequent specific drug prone to the DTPs followed by spiranolactone (14.52%), enalapril (6.45%) and Furosemide (6.45%). This was different from the result of a study in Norway in which the drugs most frequently prone to DTP were Warfarin, digitoxin and prednisolone [22]. This difference might be due to variation in the type of diagnosed diseases and utilized drug therapies.

A number of studies reported that involvement of clinical pharmacists in patient care in the inpatient hospital settings resulted in safer and more effective medication use through identification, resolution and prevention of drug therapy problems [25–31]. As a result, it is recommended to provide pharmaceutical care service at DRH in order to minimize the occurrence of DTPs.

As limitation, this study didn’t determine the severity and outcomes of DTPs. Information on past medication history was mainly obtained from the patients or attendants as documentation was scanty. Identification of an ADR was based on clinical assessment made by physicians. Thus the findings might be an underestimate of the number of ADRs. When DTPs were identified, the physicians were informed for ethical reasons and this may have further lead to reduction of subsequent number of DTPs. Critically ill patients requiring ICU admission were excluded from the study which leads to exclusion of complicated cases. Additionally, Convenience sampling restricted to a 3 month study period may lead to failure in capturing probable seasonal variations.

## Conclusion

Three out of four patients experienced at least one drug therapy problem during their hospital stay in the medical ward of Dessie referral hospital, with the most commonly observed DTP being no drug therapy prescribed for a condition requiring drug treatment. Most of the drug therapy problems were occurred during the treatment of cardiovascular disorders, and ceftriaxone was found to be the most frequent drug involved in the DTPs.

## Abbreviations

ACEI: Angiotensin converting enzyme inhibitor
ADR: Adverse drug reaction
CNS: Central nervous system
DRH: Dessie referral hospital
DRP: Drug related problem
DTP: Drug therapy problem
GI: Gastrointestinal
JUSH: Jimma University specialized hospital
NSAID: Nonsteroidal anti-inflammatory drug
OI: Opportunistic infection
RTI: Respiratory tract infection
RVI: Retroviral infection
